# Reversed-phase allergen microarrays on optical discs for multiplexed diagnostics of food allergies

**DOI:** 10.1007/s00604-023-05756-5

**Published:** 2023-04-03

**Authors:** Luis A. Tortajada-Genaro, Natalia Casañ-Raga, Salva Mas, Ethel Ibañez-Echevarria, Sergi Morais, Ángel Maquieira

**Affiliations:** 1 Instituto Interuniversitario de Investigación de Reconocimiento Molecular Y Desarrollo Tecnológico (IDM), Universitat Politècnica de València, Universitat de València, Camino de Vera S/N, 46022 Valencia, Spain; 2 Departamento de Química, Universitat Politècnica de València, Valencia, Spain; 3 Unidad Mixta UPV-La Fe, Nanomedicine and Sensors, IIS La Fe, Valencia, Spain; 4 Hospital Universitari I Politènic La Fe, Servicio de Alergología, Avinguda de Fernando Abril Martorell, 106, 46026 Valencia, Spain

**Keywords:** Food allergy,Micro-immunoassay, Immunoglobulin E, Optical biosensor, Protein array

## Abstract

A high percentage of the population suffers from multiple food allergies justifying  the importance of reliable diagnostic methods. Single-analyte solutions based on the determination of specific immunoglobulins E (sIgE) are safe and fast but are generally time-consuming and expensive. Thus sustainable microanalytical methods that provide multianalyte profiling information are highly demanded. This work presents the in vitro biosensing of specific IgE levels based on a reversed-phase allergen array. The approach consists of optical biosensing supported by direct multiplex immunoassays and on-disc technology. It identifies 12 sIgE associated with food allergies in a single analysis with a low serum sample volume (25 µL). After processing captured images, specific signals for each target biomarker correlate to their concentration. The assay analytically performs well with 0.3 IU/mL and 0.41 IU/mL as the detection and quantification limits in serum, respectively. This novel method achieves excellent clinical specificity (100%) and high sensitivity (91.1%), considering the diagnosis obtained by clinical history and ImmunoCAP analysis. The results demonstrate that microanalytical systems based on allergen arrays can potentially diagnose multiple food allergies and are easily implemented in primary care laboratory settings.

**Graphical Abstract:**

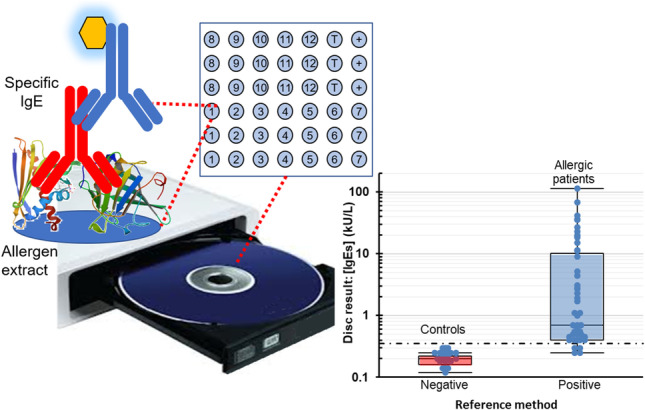

**Supplementary Information:**

The online version contains supplementary material available at 10.1007/s00604-023-05756-5.

## Introduction

Food allergy is a chronic disease caused by skin exposure, inhalation, or ingestion of particular foods [1]. Allergenic compounds can cause adverse reactions, mainly type I hypersensitivity or immunoglobulin IgE-mediated reactions, with diverse negative symptoms. As its prevalence is increasing, food allergy is an acknowledged substantial public health burden [2, 3]. Although several studies point out that a high percentage of patients are allergic to a single food, many people have multiple food allergies [4]. From a holistic approach to food allergy, it is necessary to correctly identify allergic patients to design prevention actions and reduce associated health costs accurately.

In vitro quantitative measurements of specific IgE antibody levels have been increasingly applied in the past decade to overcome the drawbacks of skin prick tests [4]. Several immunological methods have been developed, but they are generally limited to a single allergen and require large volumes of serum, their analytical performance is poor, or they are performed on large automated equipment [5]. In recent years, advances in high-throughput molecular diagnostics have been incorporated into clinical facilities [6, 7]. Nevertheless, advanced research into microsystems and biosensing is required, especially for reliable diagnoses for patients with multiple food allergies [8]. Hence, the current analytical challenges include developing multiple-analyte assays with improved sensitivity, small sample volumes, and short analysis times.

Protein microarrays are essential for multiplexed analyses in different diagnostic areas [9]. They are highly miniaturized tools with a panel of capture molecules immobilized in a small area (µm^2^). Such assay systems require only minute amounts of sample and reagent volumes (nL-µL). Several authors have developed chip assays in a miniaturized array format, which focuses on simultaneously identifying human diseases based on detecting specific biomarkers. The conventional food allergies approach is a forward-phase capturing array that immobilizes each allergen (isolated protein or recombinant protein) on a glass slide [10]. Later, the sample is incubated on the chip, and specific biomarkers are recognized. Finally, a fluorescence scanner is typically used to register the spot intensities produced by fluorophores-stained antibodies. Silicon chips coated with a functional polymer and three-dimensional gel-based microchips have also been proposed as alternative assay substrates [11, 12]. To date, a microarray for IgE antibody testing is commercially available called the Immuno Solid-phase Allergen Chip (ImmunoCAP ISAC, ThermoFisher Scientific). This method is considered the gold standard in food allergy diagnoses from serum.

Modern diagnoses require a parallel (multiplex) highly sensitive analysis that allows the simultaneous determination of different biomarkers in a single assay supported by an affordable portable technology [13]. Microfluidic chip platforms have been developed to miniaturize IgE-based assays in a forward-phase format [14, 15]. However, the chips fabricated by soft lithography are connected to a complex external assembly to control the flow of samples and reagents, and they also require a fluorescent scanner.

An essential drawback of forward-phase arrays is the limited availability of the specific allergenic protein in its recombinant or natural origin form. Another disadvantage lies in the Ig-mediated adverse response sometimes associated with the simultaneous effect of several proteins.

A reverse-phase array is a relevant protein abundance microarrays category [16]. This technique comprises an immobilized cellular or protein-based lysate [17]. The common substrate is nitrocellulose due to its high protein-binding capacity (e.g., 0.25 mg/mL total protein). Assays can also be constructed in multipad or sector formats, which comprise numerous nitrocellulose pads on one glass slide or 96-well plates, respectively [16]. In the allergy field, the proposed microarray is based on protein extracts of food components [18]. Glass slides coated with a nitrocellulose polymer have been used to capture proteins in a noncovalent mode using a four-color fluorescence scanner as the detector. The reported results indicate good discrimination between challenge responders in atopic and nonatopic individuals, and between poly- and mono-specific IgE responders. Another feasible approach involves a reverse-phase format in a nitrocellulose membrane and a colorimetric scanner [19]. These methods enable improved IgE detection, but only in laboratories with specialized infrastructure and bench instruments.

Herein, we present a reliable, affordable assay to determine serum sIgE levels based on protein extracts and digital versatile technology (DVD). This consumer electronics device can be practically performed to develop compact biosensing systems intended for the point-of-care testing of several biomarkers [20, 21]. The methodology involves similar steps to slide microarrays, but discs are polymeric supports used to carry out assays with small volumes of reagents, and the disc drive is the imaging system. Optoelectronic technology has been successfully applied in clinical diagnostics, including the immunorecognition of allergenic proteins in food samples for safety-quality control [22]. In the present study, the novelty lies in developing a method to quantify sIgE in serum samples by combining the immobilization of allergenic proteins (forward-phase) and food lysates (reversed-phase) on the same disc. The aim is to demonstrate the feasibility of low-cost allergen microarrays for the optical multiplexed detection of a panel of 12 specific IgEs associated with food allergy in minimal-specialized clinical laboratories. To deliver a fully integrated assay on a disc with several samples, multiple steps of this microimmunoassay have been developed, such as probe immobilization, sample incubation, washing, staining, and reading.

## Material and methods

### Materials

The spotting solution contained 50 mM carbonate buffer at pH 9.6 and 1% glycerol (v/v). Phosphate-buffered saline (PBS) was prepared at 0.8% NaCl, 0.02% KCl, 0.02% KH_2_PO4, 0.3% Na_2_HPO_4_, and pH 7.5. The washing buffer was PBS with 0.05% Tween-20 (PBST). The proteins from food sources were provided by ALK-Abelló (Spain), Leti Pharma (Spain), Roxall (Germany), and Allergy Therapeutics (UK). The protein extracts included wheat (*Triticum aestivum*), barley (*Hordeum vulgare*), kiwi (*Actinidia deliciosa*), oily fish, prawn (*Parapenaeus longirostris*), peach (*Prunus persica*), sesame (*Sesamum indicum*), cow’s milk (*Bos taurus*), chicken egg (*Gallus gallus*), walnut (*Juglans regia*), peanut (*Arachis hypogaea*), and pistachio (*Pistacia vera*). The pure proteins were profilin, ovalbumin, beta-lactoglobulin, lipid transporter proteins from peach (LTP), and casein.

The employed primary antibodies were monoclonal anti-human IgE antibody and mouse anti-human IgE monoclonal antibody (Ingenasa-Eurofins, Spain). The polyclonal anti-human IgE antibody (pAb) conjugated to horseradish peroxidase (HRP) was employed (Dr. Fooke, Germany). The positive controls were human IgE (Abcam) at 10 ng/µL. The negative control was human serum albumin at 10 ng/µL (HSA, Sigma-Aldrich). The 3rd international standard for serum IgE, 11/234, was from NIBSC (Hertfordshire, UK). Tetramethylbenzidine (TMB) substrate was a ready-to-use reagent (ep(HS)TMB-mA, SDT GmbH, Germany).

### Human samples

Serum samples were collected in red-top tubes (BD Diagnostics, Madrid, Spain) and incubated at room temperature for 60 min to induce clotting. After centrifuging at 2000 rpm for 15 min, serum was aliquoted into cryovials and stored at − 80 °C until used. The study included a cohort of 34 food-allergic patients and 21 controls. All the individuals were characterized according to their clinical history, prick-test result, and the concentration of the sIgE measured by ImmunoCAP. The participants were enrolled after providing written informed consent according to the protocols approved by the Ethics Review Board (Hospital Universitari i Politènic La Fe Valencia, Spain).

### Disc preparation

As described in the supplementary material, the dilutions of allergen proteins or food extracts (40 ng/mL) in printing buffer were spotted on digital versatile discs (DVDs). Each disc contained 14 arrays. Probes were spotted in a 6 × 7 format. Each array included 12 food extracts (three replicates per extract), anti-total IgE antibodies, and positive controls. Discs were incubated overnight at room temperature to promote surface coating by passive adsorption.

### Immunoassay protocol for IgE determination

Patients’ serum (25 µL) was mixed with 0.5% Tween-20 (25 µL) and dispensed on each disc array (14 samples per disc). After incubation for 30 min at room temperature, the disc was washed with PBST and water and dried. Next, the HRP-labelled primary antibody (anti-human IgE) in PBS at the 1/800 dilution was dispensed for 30 min, and the disc was washed as before. Colorimetric staining consisted of direct TMB substrate dispensation. After a 10-min incubation, washing with deionized water stopped the reaction. The calibration curve was prepared from WHO standards (immunoglobulin E human serum) in a human control serum at different concentrations (0.3–100 kIU/L).

### Reading

A modified DVD drive (LG electronics, DVD GSA-H42N) acquired surface images. Custom software performed the reader control and the data analysis to quantify spot signals (Figure SI.1). The scanning parameters were speed 8 × , gain 25 dB, and processing diameter 250 mm (460 pixels per spot). Details are provided in the Supplementary Material. The automated data analysis generated digital signals for each spot, such as pixel number, mean spot intensity, standard deviation of spot intensity, and local background signals. The signal-to-noise ratios were calculated as the ratio of the spot signal and the standard deviation of the background noise. The detection limit was estimated as the concentration of the correspondent immunoglobulin that modified 10% of the background signal.

## Results

### Assay principle

**Fig. 1:**
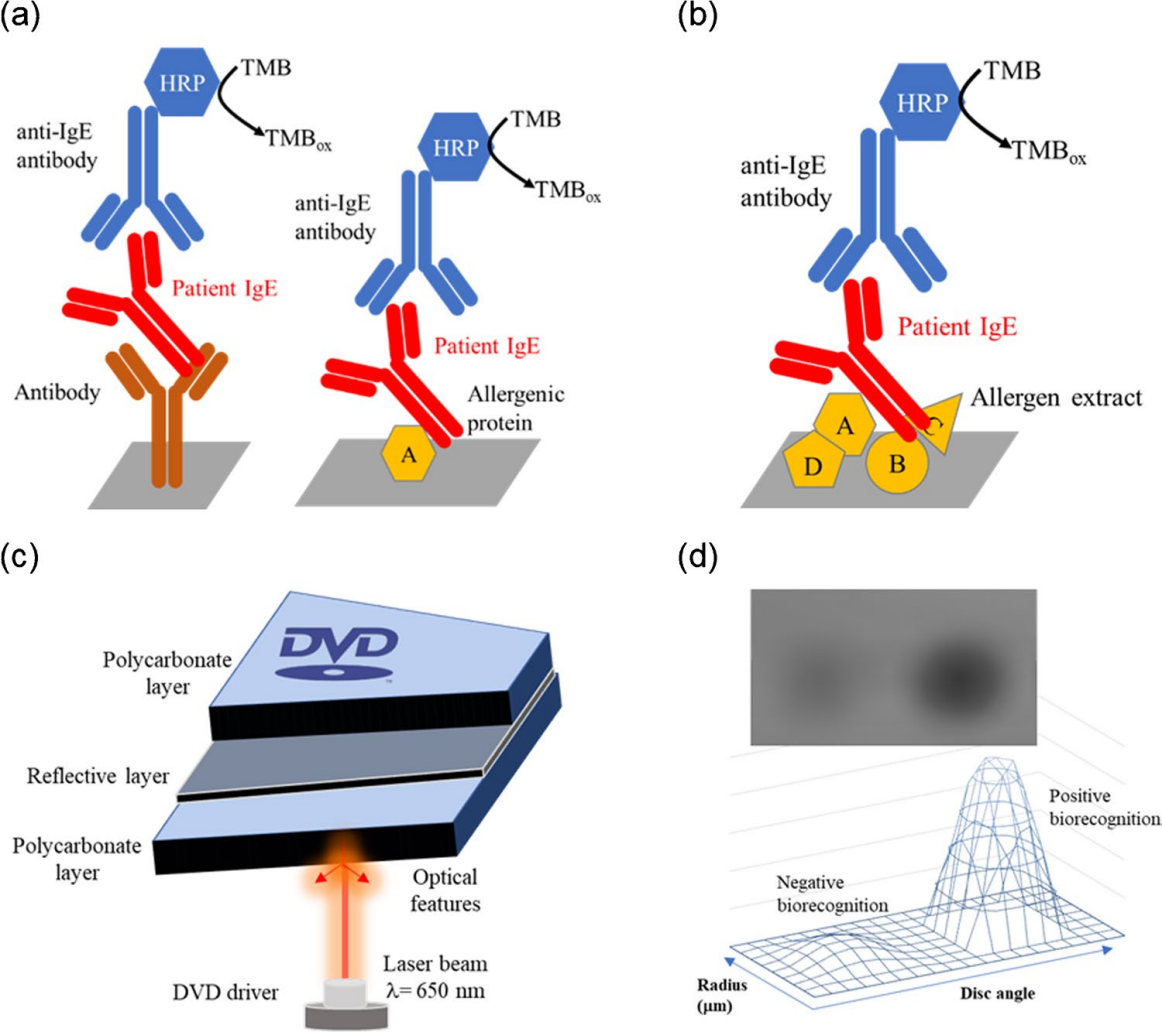
Scheme of the optical biosensing approach based on multiple immunoassays in an array format. **a** Forward-phase and **b** reverse-phase protein arrays. **c** Reading principle on the disc surface by the DVD laser beam. In the reflection mode, the intensity of the reflected beam changes in the presence of biorecognition elements on the disc surface. **d** The image corresponds to negative and positive spots, and the graph represents the optical intensity depending on disc coordinates

The proposed method uses a protein microarray designed for combining single-molecular docking and a dot-blot platform (e.g., a complex mixture of proteins). It allows the measurement of protein concentrations in a large number of biological samples simultaneously and quantitatively (Fig. 1). Specific allergenic proteins are immobilized in a forward-phase format on the sensing surface. In the reverse-phase, partially purified protein extracts from target foods are directly spotted. They are subsequently probed with patient serum containing immunoglobulins E, some associated with a specific food allergy. The biorecognition process yield is determined by quantifying the formed product on the analytical surface.

The selected detection principle is based on compact disc biosensing, a competitive technology given its low cost and portability compared to other imaging technologies. During disc scanning, the laser hits the immunoreaction product, which modifies the reflection properties of the disc surface. The optical density of the immunoreaction product is proportional to the analyte concentration.

### Optimization of sensing events

Pure proteins, protein extracts, and monoclonal anti-human IgE antibodies (positive control) were compared as probes for recognizing allergy biomarkers. The protein extracts for the in vitro assay were obtained from the reagents used for skin tests in clinical facilities. The surface chemistry that leads to probe immobilization was one of the most crucial aspects of the protein array architecture. In this study, the direct attachment of allergens by passive adsorption was approached by the native functional groups of proteins. The device was tested by measuring WHO-IgE standards at 0–50 kIU/L for calibration between the spot signal and IgE concentration. The results indicated that direct adsorption onto the activated polycarbonate surface was adequate for immobilizing antibodies, pure allergen proteins, or protein extracts, yielding sensitive recognition and optical transduction. Although probe orientation was uncontrolled, the experimental conditions allowed the recognition domain to remain unaltered, which enabled strong binding to the target molecule and to report detectable spot intensity. This study was the first time that protein extracts were used as recognition elements in a DVD-based biosensing system.

The amount of immobilized allergen was critical for the method’s analytical performance. The optimal concentration of each coated allergen protein or extract was selected by checkboard titration assays (Figure SI.2). Other variables were studied, such as developing reaction (Figure SI.3) and matrix effect (Table SI.1). The recognition event was displayed using anti-human IgE conjugated to peroxidase to allow fast colorimetric detection after adding a substrate. The experiments demonstrated that the diluted serum in buffer solution (1:1) and the calibration standards in the diluted control serum (free of IgE) provided satisfactory results to determine protein levels accurately. Analytical performances were similar to those reported for single-allergen methods [5–7]. Most current techniques need a blocking step to reduce the nonspecific binding of serum components. However, the chemical properties of the thermoplastic materials of discs avoided this step, and the platform can potentially be a high-throughput proteomics platform compatible with the typical lysates used in published reverse-phase protein arrays [9].

### Multiplexing capability

The following research focused on assessing the capability of detecting several specific IgEs simultaneously by our optoelectronic technology as a transductor. This type of test is applicable for multi-allergic patients and scenarios that require high working capacity, i.e., many samples simultaneously. However, multitarget assays can present cross-reactivity, and assay selectivity must be confirmed before being applied [7, 23].

**Fig. 2:**
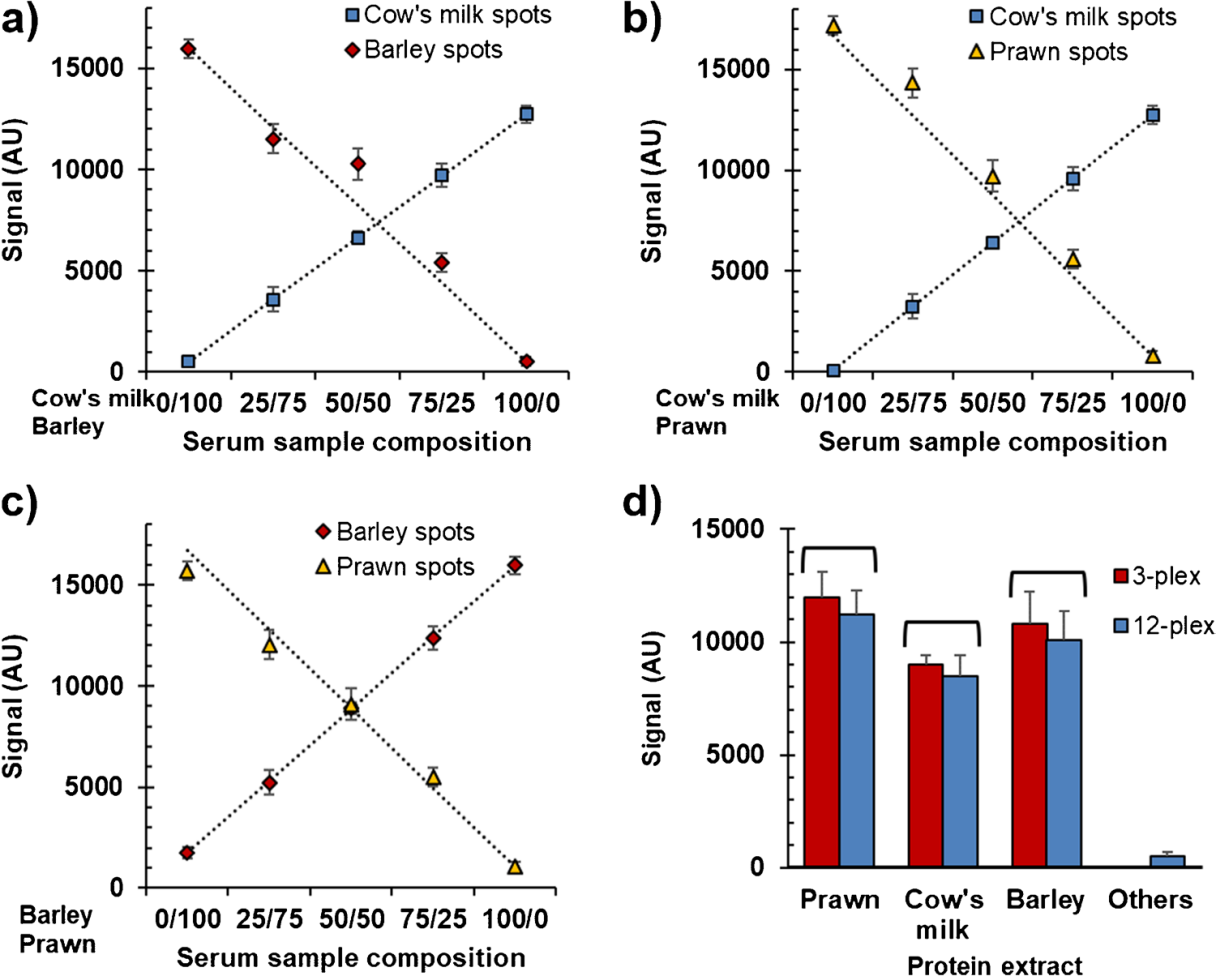
Evaluation of multiplexing capability based on spot intensities for binary mixtures (**a**–**c**) and the ternary mixture (**d**). Serum samples: Confirmed patients allergic to the correspondent food allergen. Probes: extracts from prawn, barley, and cow’s milk. Replicates = 4 spots × 3 samples (mean ± standard deviation). Signal statistics, *p*-values of *t*-test: 0.42 (prawn), 0.43 (cow’s milk), and 0.56 (barley); *p*-values of *F*-test: 0.99 (prawn), 0.34 (cow’s milk), and 0.87 (barley)

In the first approach, we developed a triplex test capable of determining specific IgE associated with allergies to prawn (*Parapeanuns longirostris*), barley (*Hordeum vulgare*), and cow’s milk (*Bos taurus*). The prepared discs analyzed the artificial multi-allergic pooled sera generated from the mixtures of samples from the patients with a history of reactivity to these allergens. Qualitatively, the recognition profiles were studied to find that spots presented a significantly different response from the background (Figure SI.4). The results showed high assay selectivity because the different specific IgEs recognized only their corresponding immobilized allergen. Thus, good discrimination between mono-specific and poly-specific IgE responders was achieved. Quantitatively, the linear relation between the mean spot intensities and the sample proportion from a patient allergic to a specific allergen was excellent (Fig. 2). The mean recovery percentages were 100–106% for the three allergens.

Finally, experiments examined its capability as a high-multiplexing test because the analytical performances associated with a particular biomarker could depend on the nature and number of targets that had been simultaneously assayed [10, 14, 20]. The method was extended from a panel of three allergens to 12 by following the same strategy of immobilizing protein extracts in a microarray assay. All the studied formats reported specific responses, and spot signals were comparable, even after increasing the number of extracts spotted on the same array (*t*-test: *p*-value > 0.05, *F*-test: *p*-value > 0.05). Hence, the results verified that the developed microsystem could be employed to simultaneously determine several targets in a single run and with a small serum volume (25 µL).

The following experiments estimated other the assay’s analytical performances. IgE standards were incubated for sensitivity evaluation in a control serum (up to 100 kIU/mL). The detection limit was 0.30 ± 0.04 kIU/L (0.72 ± 0.10 ng/mL), calculated from the recorded signals of controls and standards. Reproducibility was obtained from replicated assays on the same day and between different days. The relative standard deviations for the sIgE concentrations were 2–7% (intra-day) and 10–25% (inter-day). Considering published studies about biosensing methods developed for determining targets for food allergies, the results were satisfactory (Table SI.2). In conclusion, the developed approach presents the appropriate analytical benefits for its application to determine IgE levels in serum samples to diagnose multiple food allergies.

### Application: in vitro diagnosis of food allergies based on IgE levels

**Fig. 3:**
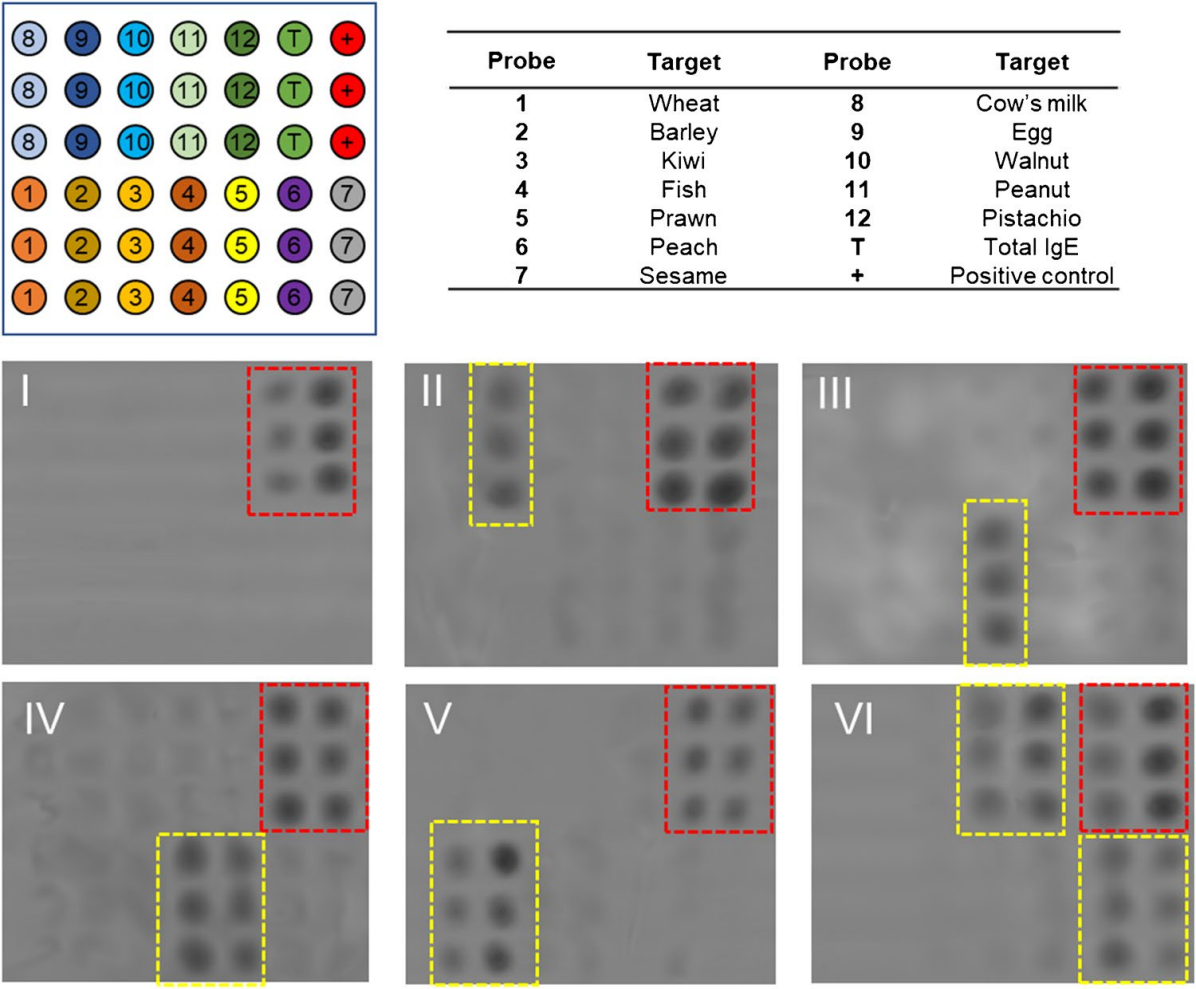
Assay in the 12-plex format. Array layout scheme (Top). Examples of the array images obtained for a serum sample from the recruited individuals (Bottom). I, nonallergic; II, allergic to egg allergy; III, allergic to fish; IV, allergic to prawn and fish; V, allergic to wheat and barley; VI, allergic to peach, sesame, peanut, and pistachio. Red rectangle, total IgE and control spots. Yellow rectangle, detected allergens

The developed system’s capability to identify allergic patients was examined by evaluating the response pattern against the panel of allergens. The first objective was to establish which specific IgEs were present in each serum by focusing on preventing misdiagnosis of allergies and guiding food avoidance [2, 8]. Multiplexed immunorecognition was performed in the microarray format and included 12 specific food allergies (Figure SI.5). Precise recognition profiles were obtained, which allowed the easy identification of atopic individuals (Fig. 3). The positive spots for total IgEs and controls were observed for all the patients. Signal intensities higher than the detection threshold were only observed for certain protein extracts, which indicates the culprit food allergy.

**Fig. 4:**
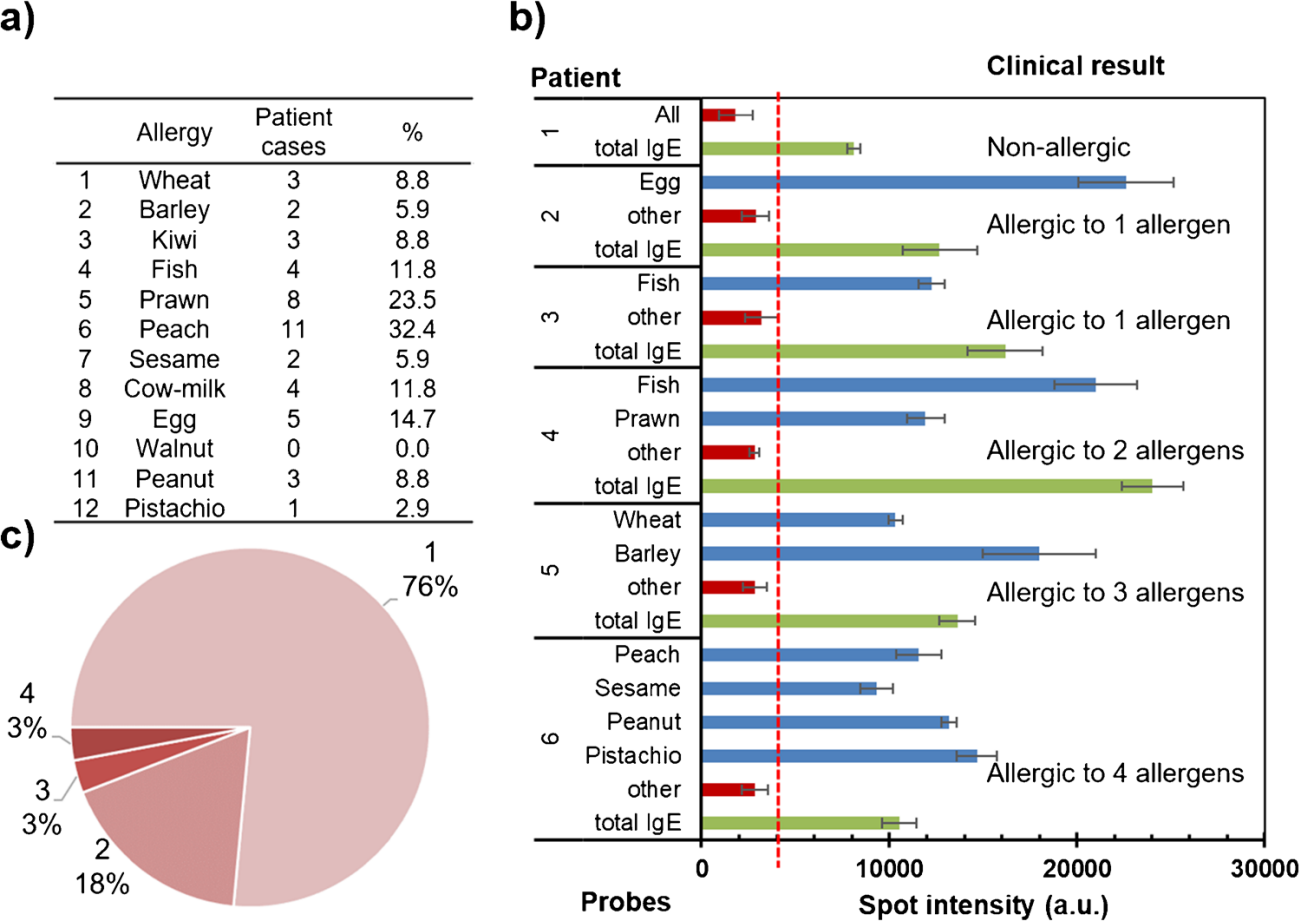
Assay in the 12-plex-format applied to analyze 55 patient sera samples. **a** Number and percentage of positive cases for the studied food allergies. **b** Bar chart of the spot intensities for six representative patients and clinical results based on the detected probes. Replicates = 3 spots × 3 samples (mean ± standard deviation). The red line indicates the signal associated with the detection limit. **c** Population percentage classified by considering the number of detected allergens per patient

In our pilot study (55 samples × 12 allergies), 30 individuals were classified as positive for at least one allergen. The identified allergens were 41 cases, and 11 types of food allergies were found (Fig. 4a). The most detected food allergy was peach (11 patients), and the least was walnut (1 case) and pistachio (1 case). From the clinical point of view, both correct management and diet control also require accurately identifying patients with single or multiple food allergies [24]. As shown in Fig. 4b, the developed assay detected positive responses for multiple extracts in several patients. The percentage of the total identified atopic individuals was 27%, and the maximum number was four food types (Fig. 4c). In most multi-allergic cases, the positive responses corresponded to extracts from related families, such as cereals or nuts. As these foods share homologous proteins, IgE-mediated sensitization to one food can result in clinical reactivity to food families.

Future variants of this method can be designed for target food families by changing the panel of immobilized extracts on the analytical surface. This tool might help to guide food avoidance. It can also support the diagnosis of allergies that results from hidden allergens, i.e., ingredients not declared on product labels, and might unexpectedly reach end food products through several routes, such as accidental contamination. The advanced formats of analytical devices can be incorporated to improve method feasibility and working capacity, i.e., the number of samples per assay or assay automation [21, 24–26].

**Fig. 5:**
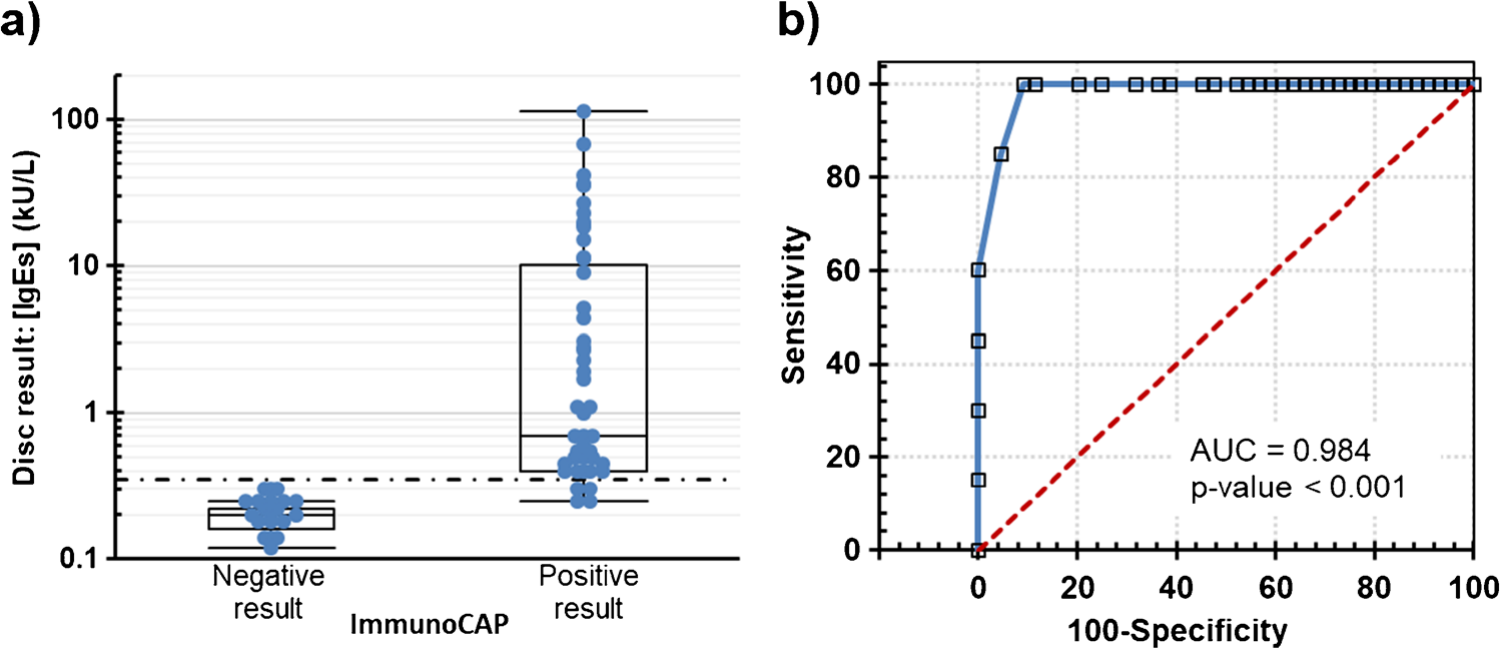
In vitro diagnosis of food allergies by DVD-based technology. **a** Populations of IgE levels from serum samples according to disc and ImmunoCAP results: negative (21 nonatopic individuals) and positive (34 patients, 44 allergens). **b** ROC curve

The following experiments quantitatively determined specific IgEs levels in serum (blind study). The validation of the approach consisted of making a comparison to ImmunoCAP, which is considered the gold standard in vitro diagnostic test for food allergies [2]. The historical threshold (0.35 kIU/L) was used as a cut-off for positive and negative results in ImmunoCAP. The comparative populations of the measured IgE antibody levels are shown in Fig. 5a. A good agreement was found when classifying patient groups (21 negatives and 30 positives with 41 allergens), but there were some discrepancies (4 allergens) (Table SI.3). In these cases, the reported values were 0.35–0.45 kIU/L (low positive) for ImmunoCAP and 0.20–0.30 kIU/L (negative) for the biosensing method. Receiver operating characteristic (ROC) analyses examined the ability to confirm sensitization to food ingredients (Fig. 5b). Our approach exhibited good clinical prediction, with an area under the curve (AUC) of 0.984 ± 0.010 (*p* < 0.001), 91.1% sensitivity, and 100.0% specificity. This biomarker information might contribute to correct diet selection, although cross-reactivity to homologous proteins can yield positive test results in a clinically tolerant patient.

Likewise, the novel in vitro method is a low-cost, ubiquitous, and portable device with an accurate, sensitive, and simple assay (12 allergens, 25 µL of sample, 90 min). Thus, it is an appealing approach to run point-of-care tests compared to current technologies [2, 5, 13, 23] (Table SI.4).

## Conclusions

This paper demonstrates that microanalytical systems have the potential to diagnose diseases supported by the quantification of biomarkers. A reversed-phase allergen assay is a reliable analytical system to determine sIgE in a serum sample because these probes can recognize the intrinsic variety of allergic responses compared to single-protein approaches. An array-based method is an up-and-coming in vitro diagnostic tool for effectively identifying patients with adverse responses to multiple food components. This study proves that biochips arrays containing a set of immobilized allergens are a feasible solution for detecting 12 food allergies in a single experiment and with a small sample volume.

Combining optical biosensing and reverse-phase protein arrays should save time and costs when an accurate allergy diagnosis is required. The current research reports that an appealing solution is a microanalytical multiplexed assay supported by compact disc technology (disc, reader, software) as a portable, simple, and cost-effective sensing device. The results confirm that sensitive detection is achieved thanks to the high signal-to-noise ratios provided by the optical biosensing system. Thus, our method affords quantitative information by not only enabling health professionals to define the IgE profile better for each patient but also to improve the identification of an adequate therapeutic strategy.

In short, using allergen extracts to detect and quantify specific IgEs in the array format has advantages over most current methods supported by highly purified allergens and recombinant allergens in a single format. Hence, the impact of this technology is its easier identification of multiple food allergies.

## Supplementary information

Below is the link to the electronic supplementary material.

Supplementary file1 (PDF 1056 KB)
