# Supplementary material for: Reversed-phase allergen microarrays on optical discs for multiplexed diagnostics of food allergies
Source: Mikrochim Acta. 2023 Apr 3;190(5):166. doi: 10.1007/s00604-023-05756-5 (PMC10070211; doi:10.1007/s00604-023-05756-5)
Supplement: Supplementary file 1 — Supplementary file1 (PDF 1056 KB) [file 604_2023_5756_MOESM1_ESM.pdf]

Reversed-phase allergen microarrays on optical discs for multiplexed diagnostics of food allergies

Luis A. Tortajada-Genaro[a,b,c*], Natalia Casañ-Raga[a], Salva Mas[a], Ethel Ibañez-Echevarria[d], Sergi Morais[a,b,c], Ángel Maquieira[a,b,c]

[a] *Instituto Interuniversitario de Investigación de Reconocimiento Molecular y Desarrollo Tecnológico (IDM), Universitat Politècnica de València, Universitat de València, Camino de Vera s/n, 46022 Valencia, Spain.*

[b] *Departamento de Química, Universitat Politècnica de València Valencia, Spain*

[c] *Unidad Mixta UPV-La Fe, Nanomedicine and Sensors, IIS La Fe, Valencia, Spain*

[d] *Hospital Universitari i Politènic La Fe, Servicio de Alergología, Avinguda de Fernando Abril Martorell, 106, 46026 València, Spain*

E-mail: luitorge@qim.upv.es

TABLE OF CONTENTS

1. Preparation of DVD-based chip

2. Description of the device

Figure SI.1. Scheme of the device.

3. Assay optimization

Fig. SI.2. Immobilization yield.

Figure SI.3. Spot intensities depending on primary antibody dilution and amount of specific IgE.

Table SI.1. Matrix effect.

Figure SI.4. Recovery results (%) of the pooled serum samples.

4. Analytical performances

Table SI.2. Comparison of biosensing methods employed for food-specific IgE determination

5. Application

Figure SI.5. Scheme of microimmunoassay for the analysis of patient samples.

Table SI.3. Results for allergic patients from serum samples

Table SI.4. Examples of commercialized technologies for the detection of specific IgEs.

1. Preparation of DVD-based chip

Bulk digital versatile discs (DVDs) were purchased from MPO Iberica (Madrid, Spain). Discs were first conditioned by gentle ethanol washing, water rinsing, and dried by spinning for 1 min at 800 rpm. Dilutions of allergen proteins (40 ng/μL) or food extracts were prepared in printing buffer: 50 mM carbonate buffer at pH 9.6 and 1% glycerol (v/v). Spotting was performed using a liquid dispenser robot (Biodot AD1500, Irvine, CA), depositing 40 nL of probe solution at 25 ºC and 80 % relative humidity. The size of the spots was 500±10 µm. Each disc contained 14 arrays, and the layout depended on the experiment. For the diagnostic immunoassay of patient serum, probes were spotted in a 6x7 format per array and 20 arrays per DVD. The distance between flanking spots was 1.5 mm. Hence, each array has three replicate spots corresponding to twelve food extracts (three replicates per extract), anti-total IgE antibodies, and positive controls. Discs were incubated overnight at room temperature, promoting the surface coating by passive adsorption on the DVD substrate. Thus, the primary immobilization strategy was based on a hydrophobic bonding between the polycarbonate surface and non-polar amino acid residues in proteins, such as alanine, valine, leucine, isoleucine, phenylalanine, tyrosine, tryptophan, proline, and methionine. A blocking step was not required. The discs were stored in a sealed disc box at 4 ºC until use. Experiments confirmed that the probes were active for at least 30 days.

2. Description of the device

The reading principle is based on using disc and disc drives to monitor molecular biorecognition events. DVDs are the analytical platform that supports the immunoassay in a microarray format. A modified DVD drive is the optoelectronic technology applied in the transduction process (**Figure SI.1**).

The reader electronics comprise a commercial DVD drive (LG electronics, DVD GSA-H42N), custom-built hardware, and the commercial data acquisition board (DAQ) USB-2527 (Measurement Computing Corporation, Massachusetts, USA). The biosensor includes the photodiode S3588 (Hamamatsu Photonics K.K., Hamamatsu, Japan) as the main component of the detection board. The optical pickup unit (OPU) generates the laser beam at 650 nm, passing through the optically transparent disc-shaped platform. The photodiode detects the transmitted light and induces modulated signals that correlate the power absorption of the spots according to the analyte concentration. The photodiode has a sensitivity of 0.46 A W–1 at 650 nm and a spectral response range from 340 nm to 1,100 nm. The best reading speed was 8x (equivalent to 10,820 kB per second). Considering biosensing requirements, this speed provided an excellent captured image with the lowest reading time.

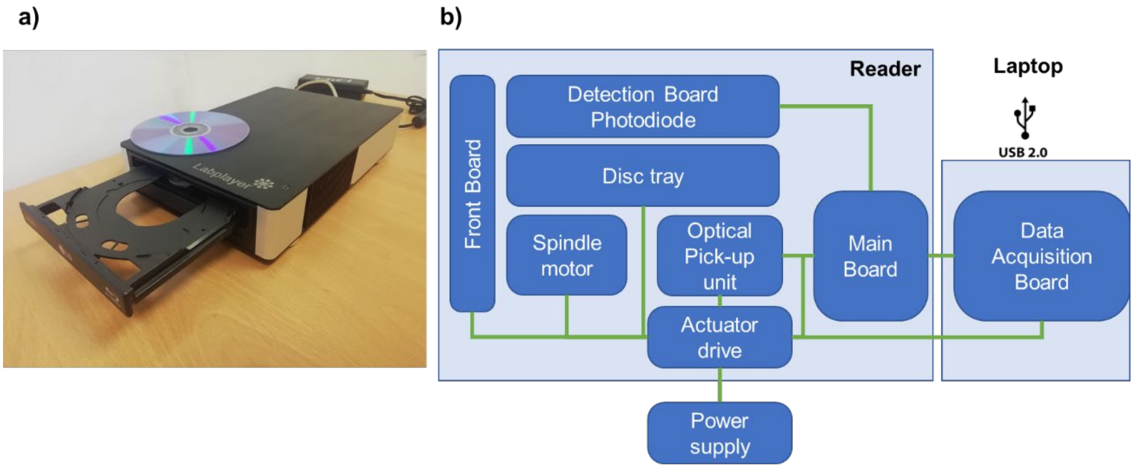

Figure SI.1.: a) Image of the device for multiple micro-immunoassays. Disc dimensions: diameter 12 cm and thickness 1.2 mm. Reader dimensions: 17.5×26.5×5 cm. b) Scheme of the reader, containing the optomechanical components, mechanical structure, and configurations of the DVD drive and their modifications, such as detection board photodiode and data acquisition board.

A black adhesive (10×1 mm), glued on the DVD surface close to the first array, was used as a trigger for limiting the detection radius. During the reading, the laser beam (λ= 650 nm) scans the detection zone, being able to correlate the variations of the intensity of the transmitted beam with the optical density of the spot, which is proportional to the concentration of a specific analyte. Subsequently, the transmitted signals were obtained from the photodiode and digitized using the DAQ. The custom software, which works under Windows (W10) and is written in Microsoft Visual C ++, generated an image for each microarray in jpeg-format. Each image corresponds to one of the microarrays of the disc. An image/data analysis algorithm divided the image into a set of objects that identified each one inside a position of the array. In the current study, the array included 7×7 spots from fourteen probes and three replicates. The data analysis generated the digital signals and quality parameters (concentration, error calculation, and quality controls) for each spot by subtracting the local background from the mean signal value of the spot.

References

1. Morais, S., Tortajada-Genaro, L. A., Arnandis-Chover, T., Puchades, R., & Maquieira, A. (2009). Multiplexed microimmunoassays on a digital versatile disk. Analytical chemistry, 81(14), 5646-5654.

2. Morais, S., Puchades, R., & Maquieira, Á. (2016). Disc-based microarrays: principles and analytical applications. Analytical and bioanalytical chemistry, 408(17), 4523-4534.
3. Mas, S., Badran, A.A., Juárez, M.J., Hernández-Fernández de Rojas, D., Morais, S., Maquieira, A. Highly sensitive optoelectrical biosensor for multiplex allergy diagnosis. Biosensors and Bioelectronics 166, 112438.
4. Teixeira, W., Pallas-Tamarit, Y., Juste-Dolz, A., Sena-Torralba, A., Gozalbo-Rovira, R., Rodríguez-Díaz, J., Navarro, D., Carrascosa, J., Gimenez-Romero, D., Maquieira, A., Morais, S. (2022). An all-in-one point-of-care testing device for multiplexed detection of respiratory infections, Biosensors & Bioelectronics 213, 114454

3. Assay optimization

Coating concentration. The optimal concentration of each coated allergen protein or extract was selected by checkboard titration assays, employing allergen standards ranging from 0 to 50 kIU/mL. **Figure SI.2** shows that the recorded signal increase follows a saturation behaviour, achieving a maximum related to the adsorption capacity on the analytical surface. The coating concentrations were 10 ng/µL for anti-IgE antibodies, 5-25 ng/µL for pure proteins, and 50-500 ng/µL (total protein) for food extracts in carbonate buffer. The selection criteria were high sensitivity, reproducibility, and signal-to-noise ratio (S/N > 25). As expected, protein extracts showed lower responses than pure allergen protein at the same concentration. Considering the selected immobilization strategy, this behaviour is associated with the relative concentration of target proteins compared to total proteins.

Staining reaction. The recognition event was displayed using anti-human IgE conjugated to peroxidase to allow fast colorimetric detection after adding a substrate (**Figure SI.3**). High reproducible signals were obtained using 1/800 as a dilution factor for the antibody developer. The amount of coloured deposit onto the chip surface was proportional to the target concentration, enabling quantifying the protein levels in a high-throughput format.

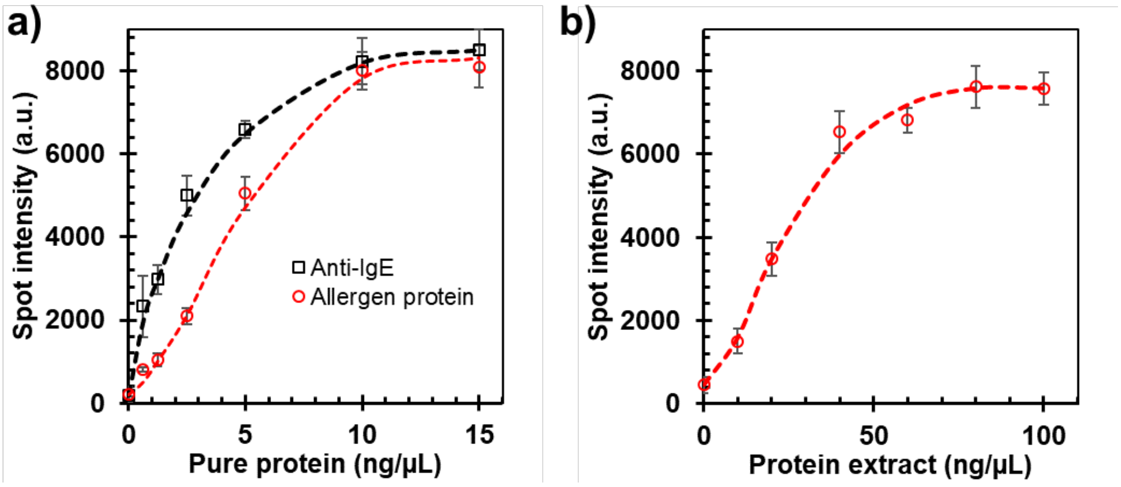

Fig. SI.2. Immobilization yield: (a) forward-phase array based on specific antibody or allergen protein (lipid transporter protein) and (b) reverse-phase array (wheat). Serum samples from allergic patients: peach (a) and wheat (b). Replicates: 5 (mean±standard deviation).

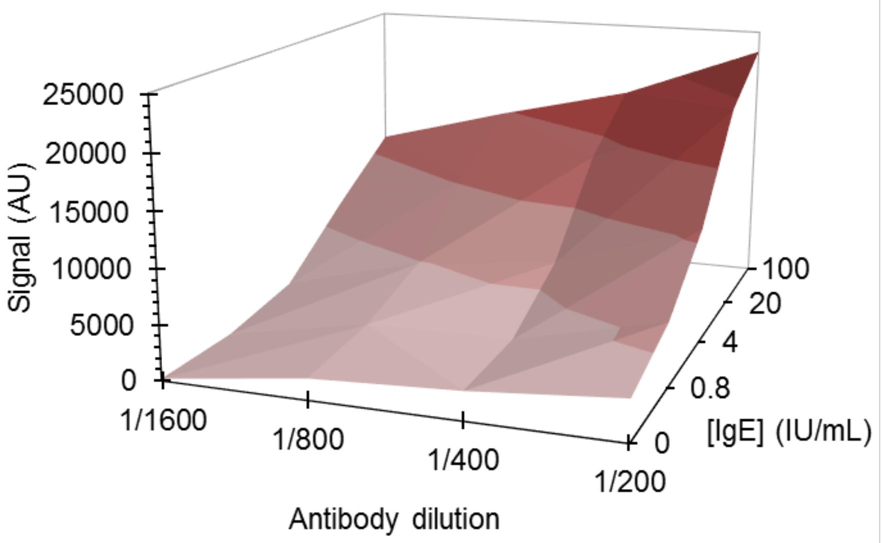

Figure SI.3. Spot intensities depending on primary antibody dilution and amount of specific IgE. Replicates = 3.

Matrix effect. For an accurate determination of immunoglobulins in clinical samples, the dilution of patient serum in different solvents was examined (**Table SI.1**). Components of the serum may lead to alterations in the registered array signals, as reported in multifactor analysis (p = 0.0165). The signal decreased 1.3 folds in undiluted serum compared to saline buffer solutions, increasing the detection limit to 0.18 kIU/L. But, experiments demonstrated that the diluted serum in buffer solution (1:1) and calibration standards in diluted control serum (free of IgE) provided satisfactory results for accurately determining the protein levels.

Table SI.1. Matrix effect. (a) Spot intensities (mean±standard deviation). Replicates = 3. (b) Parameters of logistic equation. A is the response at an infinite concentration of analyte, B is the slope at the inflection point of the sigmoidal curve (Hill slope), and C is the concentration of analyte that corresponds to 50 % of the maximum signal

(a)

[IgE] (IU/mL)	PBST solution	Diluted serum[(1)]	Raw serum
0	200 ± 200	300 ± 200	300 ± 200
0.1	600 ± 400	700 ± 300	700 ± 300
0.8	4700 ± 1800	3800 ± 2000	3300 ± 2500
4	10900 ± 3200	7800 ± 3400	6700 ± 3600
20	14900 ± 3100	12300 ± 3200	10800 ± 3800
100	16500 ± 2700	17900 ± 2400	16700 ± 2700

[(1)]Dilution: 50% serum and 50% PBST

(b)

Parameter	PBST solution	Diluted serum[1]	Raw serum
A	20200 ± 900	20700 ± 1100	20000 ± 1400
B	0.7 ± 0.1	0.7 ± 0.1	0.7 ± 0.1
C	4 ± 1	9 ± 2	12 ± 3
R^2	0.992	0.994	0.991
LOD[(1)]	0.06	0.13	0.18

[(1)]LOD: The detection limit of each assay was calculated as the concentration that gives the 10 % of the maximum signal.

$$Signal = A - \frac{A}{1 + \left(\frac{IgE}{C}\right)^B}$$

Multiplexing capability. We developed a triplex test able to determine specific IgE associated with allergies to prawn (*Parapeanuns longirostris*), barley (*Hordeum vulgare*), and cow's milk (*Bos taurus*). The experimental design consisted of immobilizing specific protein extracts in a microarray format at a concentration of 40 ng/μL. Thus, 500 μm-diameter spots of the immobilized extract allowed the recognition of specific IgEs associated with one food, considering the plausible multi-nature of immunoglobulins for several food components, i.e., adverse response for several proteins present in the same food. Moreover, different spots simultaneously recognized specific IgEs associated with various foods. The conditions of micro-immunoassay were totally compatible with the optical resolution of the photodetector (μm level). Also, artificial multi-allergic pooled sera were generated from the mixtures of patient samples with a history of reactivity to these allergens. The collected images showed specific recognition profiles depending on the clinical profile (**Figure SI.4**). For instance, the assay discriminated between mono-specific and poly-specific IgE responders.

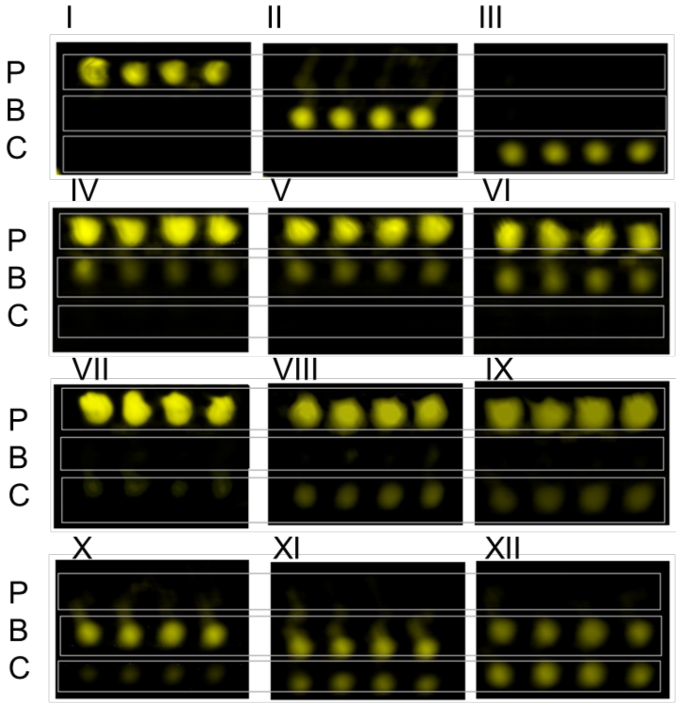

	Sample composition			%Recovery		
	Prawn	Barley	Cow's milk	Prawn	Barley	Cow's milk
**I**	100	0	0	103	-	98
**II**	0	100	0	-	100	105
**III**	0	0	100	-	100	95
**IV**	75	25	0	94	98	-
**V**	50	50	0	102	100	-
**VI**	25	75	0	112	102	-
**VII**	75	0	25	113	-	95
**VIII**	50	0	50	111	-	88
**IX**	25	0	75	108	-	94
**X**	0	75	25	-	95	110
**XI**	0	50	50	-	115	118
**XII**	0	25	75	-	113	99
			**Mean**	**106 ± 7**	**103 ± 7**	**100 ± 9**

Figure SI.4. Recovery results (%) of the pooled serum samples for the triplex assay. (Top) Array images of the twelve samples (array layout, 3 probes×4 replicates). Probes: extracts from prawn (P), barley (B), and cow's milk (C). (Bottom) Mean percentage of the recovery.

4. Analytical performances

The following table summarizes the specific features of the novel approach compared with other biosensing methods developed for identifying targets for food allergies (**Table SI.2**).

Table SI.2. Comparison of biosensing methods employed for food-specific IgE determination. Source: Trends in Analytical Chemistry, 2020, 127, 115904.

Biosensor type	LOD[1] (ng/mL)	Advantages	Drawbacks
Immunochemical	0.6-2.5	Conventional instruments<br>Low cost<br>Cost-effective<br>High throughput	Low multiplexing capability<br>Limited sensitivity<br>Large sample amount<br>Time-consuming
Electrochemical	0.005-5	Simple transducers<br>Easy to miniaturized<br>Possibility of mass production	Low number of studies<br>Low multiplexing capability
Arrays	0.4-4	Multiplexing capability<br>Low consumption of reagents<br>Small sample amount<br>High throughput	High-cost instrumentation
Nanomaterial based	20-80	Modulable properties<br>Easier miniaturization<br>Versatility	Limited sensitivity<br>Partially explored field
Label-free	190	Low consumption of reagents<br>Lower cross-reactivity<br>Fast response	Limited sensitivity<br>Sophisticated instrumentation<br>Low multiplexing capability<br>Few studies on serum
Microfluidics	1-2.5	High automatization<br>Miniaturization<br>Low consumption of reagents<br>Small sample amount<br>Portability<br>Simple for end-users	Complicated design<br>Difficult selection of materials<br>Difficulties for reagent storage
**Developed method**	**0.7**	**Multiplexing capability**<br>**Low-cost instrumentation**<br>**Small sample amount**<br>**Portability**<br>**High throughput**	**Partial automatization**

[1]LOD: limit of detection (1 ng/mL ≡ 0.417 kIU/L)

<u>References</u>

1. Morais, S., Tortajada-Genaro, L. A., Maquieira, Á., & Martinez, M. Á. G. (2020). Biosensors for food allergy detection according to specific IgE levels in serum. TrAC Trends in Analytical Chemistry, 127, 115904.

5. Application to human samples

The capability of the developed system to identify allergic patients was examined, evaluating the response pattern against the panel of allergens. **Figure SI.4** shows the DVD-based assay from serum samples and protein extracts immobilized in a microarray format. Identified allergens in serum samples are reported in **Table SI.3**. Also, the comparison with ImmunoCap technology is indicated. Finally, **Table SI.4** summarizes relevant features of commercialized technologies for detecting specific IgEs.

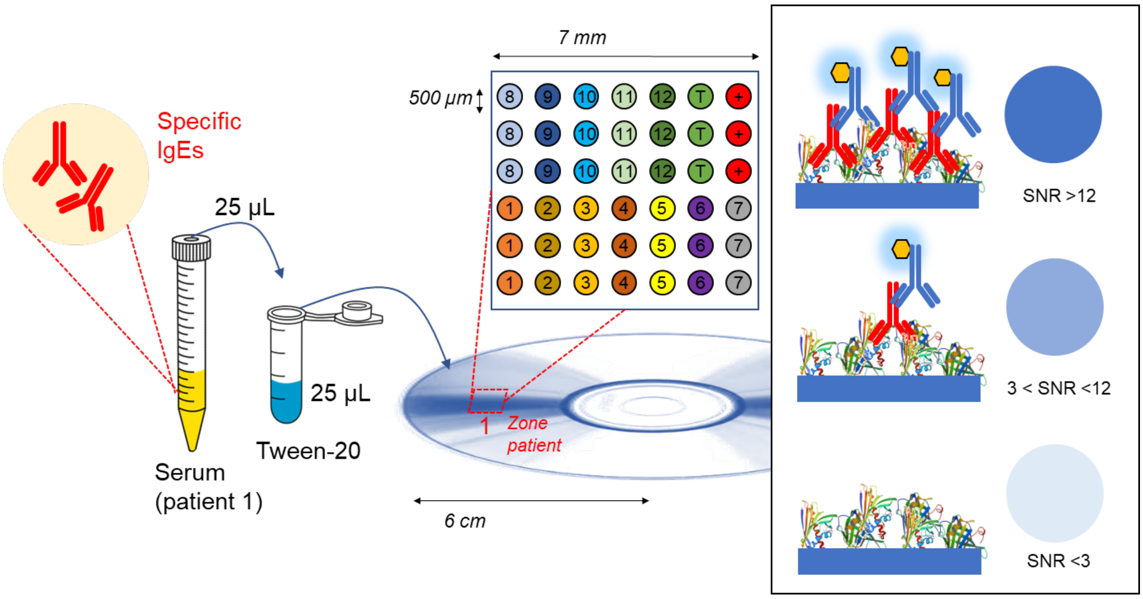

Figure SI.5. Scheme of micro-immunoassay for the analysis of patient samples. Protein extracts (probes) are immobilized on the DVD surface in a microarray format. The used reagent amount is 10-100 folds lower than other immunological techniques. Insert indicates the optical transduction of biorecognition event depending on the analyte concentration. SNR: signal-to-noise ratio.

Table SI.3. Results from serum samples

Patient number	Detected allergens	Identified allergens	Patient number	Detected allergens	Identified allergens
1	1	Cow-milk	18	2	Barley Wheat
2	1	Cow-milk	19	1	Kiwi
3	1	Peach	20	1	Prawn
4	1	Peach	21	1	Prawn
5	1	Peach	22*	0	ND (Prawn)
6	1	Peach	23	1	Prawn
7	1	Peach	24	3	Walnut Sesame Wheat
8	1	Peach	25	2	Peanut Peach
9	1	Peach	26*	0	ND (Peanut)
10	1	Peach	27	1	Egg-chicken
11	1	Peach	28	1	Cow-milk
12	4	Peanut Kiwi Peach Sesame	29	2	Prawn Egg-chicken
13	1	Oily-Fish	30	2	Barley Wheat
14	1	Oily-Fish	31	2	Kiwi Pistachio
15*	0	ND (Egg-chicken)	32	1	Cow-milk
16*	0	ND (Egg-chicken)	33	1	Prawn
17	1	Egg-chicken	34	2	Prawn Oily-Fish
35	0	ND	46	0	ND
36	0	ND	47	0	ND
37	0	ND	48	0	ND
38	0	ND	49	0	ND
39	0	ND	50	0	ND
40	0	ND	51	0	ND
41	0	ND	52	0	ND
42	0	ND	53	0	ND
43	0	ND	54	0	ND
44	0	ND	55	0	ND
45	0	ND			

* Erroneous identification: Not detected IgEs using reverse phase array technology, but identified by ImmunoCAP
ND: no detected

Table SI.4. a) Examples of commercialized technologies for the detection of specific IgEs. b) Relevant features

**Product**	**Company**	**Detection**
AdvanSure AlloScreen Max	LG Chem	Luminescence
Polycheck Allergy	Biocheck	Colorimetry
ALEX Allergy Explorer	Macro Array Diagnostics	Colorimetry
IVD Capsule Aeroallergens	Abionic SA	Fluorometry
ImmunoCap	ThermoFisher Scientific	Fluorometry

**Advantages**	**Drawbacks**
Robust technologies	Most are single-analyte assay
Detection limit 0.1-0.35 kIU/L	Expensive equipment
High selectivity and sensitivity	Test times can reach more than 3 h
Panel with a high number of allergens	Large volumes of sample 30-200 μL

References

1. Park, D. J., Lee, J., Kim, S. Y., Kwon, H. J., Lee, H. K., & Kim, Y. (2019). Evaluation of advanSure alloscreen max panel with 92 different allergens for detecting allergen-specific IgE. American Journal of Clinical Pathology, 151(6), 628-637.
2. Bojcukova, J., Vlas, T., Forstenlechner, P., & Panzner, P. (2019). Comparison of two multiplex arrays in the diagnostics of allergy. Clinical and Translational Allergy, 9(1), 1-6.
3. Pajno, G. B., Fernandez-Rivas, M., Arasi, S., Roberts, G., Akdis, C. A., Alvaro-Lozano, M., ... & EAACI Allergen Immunotherapy Guidelines Group. (2018). EAACI Guidelines on allergen immunotherapy: IgE-mediated food allergy. Allergy, 73(4), 799-815.
4. Popescu, F. D., & Vieru, M. (2018). Precision medicine allergy immunoassay methods for assessing immunoglobulin E sensitization to aeroallergen molecules. World journal of methodology, 8(3), 17.
